# Bacillus Coli Infections of the Female Urinary Tract
*A Paper read at a Meeting of the Bristol Medico-Chirurgical Society, held in the University of Bristol on 8th March, 1933.


**Published:** 1933

**Authors:** H. J. Drew Smythe

**Affiliations:** Honorary Obstetrician and Gynæcologist, Bristol General Hospital


### BACILLUS COLI INFECTIONS OF THE FEMALE URINARY TRACT.*

BY

H. J. Drew Smythe, M.D., M.S., F.R.C.S., F.C.O.G.,

*Honorary Obstetrician and Gynæcologist,*
*Bristol General Hospital.*

---

### Vulvo-vaginitis.

Primary infection of the vulva, vagina and urethra with B. Coli is not common, but cases do occur which give rise to a suspicion of gonococcal infection. The commonest type is the vulvo-vaginitis of young girls; this is limited to the external genitals, and does not involve the vagina above the hymen and rarely the urethra. The initial lesion is usually caused by trauma, either by the rubbing of tight-fitting under-clothes or by scratching from irritation, usually due to thread-worms, and infection by the B. Coli follows. On the other hand, excoriation of the parts may be caused by a highly acid urine, with subsequent Coli infection. The labia are swollen and red, and there is thick tenacious pus between the labia majora, which may be glued together. The pus is limited to the external genitals, and none can be expressed from the vagina by pressure made by a finger in the rectum, whereas in gonorrhœal cases pus can usually be expressed in this manner. The urethra is rarely

* A Paper read at a Meeting of the Bristol Medico-Chirurgical Society, held in the University of Bristol on 8th March, 1933.

infected, whereas in gonorrhœal cases it is almost invariably so. Differential diagnosis, however, rests mainly on bacteriological examination, which shows in the non-gonococcal variety a predominant growth of B. Coli, with some staphylococci.

These cases rapidly clear up under treatment, which consists in keeping the parts clean and bathing them with a weak antiseptic lotion. I have found potassium permanganate as efficacious as any, even in the non-gonorrhœal cases. After this the parts are dried with a soft towel by "dabbing," not rubbing, and then an antiseptic dusting powder is applied. If the labia tend to adhere, then an ointment may be applied between them, or lint covered with ointment placed in the natal cleft: this is especially useful at night. The initial cause must be dealt with, such as large doses of alkalis for acid urine, the treatment of the thread-worms, and the abandonment of the tight-fitting underclothes. The irritation is usually worse at night, and if this is the case then gloves should be worn by the child.

Vulvo-vaginitis of Coli origin is sometimes met with in newly-married women, and again suspicion of a gonococcal origin arises. The symptoms, however, are mild; bacteriological examination shows the presence of B. Coli in almost pure culture, and the absence of gonococci. The condition rapidly clears up under the treatment already outlined, and this rapidity of cure almost proves the non-gonorrhœal origin of the lesion.

### CYSTITIS.

Cases of acute and chronic cystitis are frequently met with in the gynæcological out-patient department, either alone or associated with pyelitis.

Large numbers of B. Coli can be passed through the bladder without setting up a cystitis, and therefore one is bound to presume a second factor as necessary to produce this condition. This factor is some local lesion of the bladder, which may be trauma or the lowering of local resistance by "chill." Trauma is the most usual cause as in catheterisation, or operation such as stripping the bladder down in performing a panhysterectomy, or again it may be due to the presence of residual urine as in the sacculated bladder of prolapse, or retention of urine caused by a pelvic tumour or the retroverted gravid uterus. Coli infection of the bladder is common after gynæcological operations, and I am sure that this is largely due to rough handling of the bladder. The infection in these cases is limited to the bladder, and does not ascend to the kidneys. The infection in B. Coli cystitis may be an ascending or a descending one; the ascending infection, in my opinion, is the most common, and I doubt whether a descending infection occurs except as an extension from a pyelitis. I do not think that a bacilluria can exist without some lesion of the urinary tract, and that the disease B. Coli bacilluria is non-existent: that it is a symptom and not a disease. I consider the presence of a large number of B. Coli in a catheter specimen as showing a lesion somewhere in the urinary tract.

Ascending infection in the female is simple: the source of infection is the anus, there is no protective bacterial flora in the urethra as in the vagina, and the urethra in the female is constantly exposed to infection; it is easily entered, is short and capacious, and it only requires some local lowering of resistance or trauma and a cystitis is set up.

In the B. Coli infection of young girls no local lesion is demonstrable, and it is probably due to the lowering of resistance by exposure to cold. The constitutional symptoms in some cases are so severe as to suggest a bacillæmia, and this view has been put forward as the origin of all Coli infections of the urinary tract. This I want to discuss later when dealing with pyelitis.

*Acute Cystitis.*

In the acute cases the symptoms complained of are frequency of micturition, both day and night, pain on passing urine, which pain is usually referred to the urethra, and is burning or scalding in character. There is a constant dull pain in the hypogastrium, often described as "dragging," and this is worse after emptying the bladder. The pain of a pure B. Coli infection, however, rarely reaches that acuteness found with other infections, and strangury is not common.

The temperature is raised, but it is never as high as in infections of the upper urinary tract, the pulse-rate is rarely increased. On examination there is tenderness over the hypogastrium, which is very marked on bimanual examination by pressing the anterior vaginal wall up against the abdominal fingers. The mouth of the urethra may be pouting and œdematous, and the vulva and inner sides of the thighs excoriated.

The amount of urine passed is copious, and if examined by the naked eye in a specimen glass there is a definite glistening sheen, very little debris, and this chiefly mucus. The reaction is highly acid: an alkaline reaction means a mixed infection, and then pus in the specimen glass is abundant. On microscopical examination B. Coli are found in abundance, a few pus-cells, bladder epithelium and a few red blood-cells. On cystoscopic examination there is swelling of the mucosa, and a certain amount of ropy mucus adherent. There is rarely ulceration except over a damaged area, which is usually at the base.

*Chronic Cystitis.*

Chronic B. Coli cystitis is rare apart from cases accompanying chronic pyelonephritis, and these are common and will be discussed later. Chronic cystitis occurs where there is some obstruction to the outflow of urine from the bladder. The most common example of this is in cases of cystocele, where, owing to the base of the bladder being below the urethral opening and the musculature stretched in this region, the bladder is not completely emptied at each act of micturition. The result is a certain amount of residual urine, which soon becomes infected via the urethra. Frequency, pain on micturition, and a sense of fullness in the hypogastrium are the symptoms which bring a large number of patients suffering from prolapse to the doctor, and very little good is done by treating the cystitis without treating the prolapse. Relief of symptoms may be obtained, but a series of relapses is inevitable.

Another frequent cause is a pelvic tumour either adherent to the bladder, dragging on it and causing sacculation, or pressing on the urethra or base of the bladder. In the last case, however, there is usually a preliminary attack of acute cystitis, and the case can only be labelled chronic in that there are a series of relapses of a less severe nature than the first.

Physical signs are few, there is no marked tenderness of the bladder, but the urethral orifice is usually pouting and red, and there may be excoriation of vulva and thighs. The underlying cause of the cystitis, usually prolapse, will, however, be found. In elderly patients these signs and symptoms may be caused by glycosuria, and the urine should always be tested for the presence of sugar. A number of cases of glycosuria have been treated for long periods under the mistaken diagnosis of chronic cystitis and vulvitis.

On cystoscopic examination the bladder mucosa is thickened and covered with mucus, there is rarely any marked contraction, but in prolonged cases, and especially in elderly women, there is atrophy of the mucosa, but rarely sacculation, though the capacity of the bladder may be considerably diminished. This is usually due, however, to a senile change rather than to an inflammatory fibrosis.

The symptoms of chronic cystitis are very slight, viz. frequency of micturition and a feeling of fullness in the hypogastrium; there is rarely pain on micturition and no constitutional disturbance. The urine examination shows much the same contents as in acute cystitis, but the bacteria are fewer in number, an occasional pus-cell, large numbers of bladder epithelial cells and very rarely a red blood corpuscle. In those cases accompanying a chronic Coli pyelonephritis the ureteric orifice on one or both sides is pouting and surrounded by a hyperæmic area of the mucosa. The treatment of acute and chronic cystitis we will discuss under that of pyelonephritis, as these two conditions demand almost identical therapy, though the latter requires more elaboration.

### PYELONEPHRITIS.

This is a very common disease of the female; it is much more frequently met with as a complication of pregnancy than in the non-pregnant state. It is a not infrequent complication of the puerperium and of gynæcological operations, though it does occur often apart from these conditions. The spread of the infection in the female is almost always a descending one; ascending infection is rare, except in cases of pelvic growths invading or obstructing the outflow of urine from the bladder, and then is a mixed infection.

The source of the B. Coli is almost certainly the bowel, but views differ as to the route taken to reach the kidney. The older view was that the infection was hæmatogenous in origin and that the primary condition was a bacillæmia, that in health the kidneys were capable of excreting the bacteria without damage to the kidney substance, but if the invasion was overwhelming, or that there was some local condition of damage or obstruction to pelvis or ureter, then pyelonephritis arises. Against this we have the considerations that B. Coli has rarely been cultured from the blood in these cases, that no other organ or structure in the body is attacked, and that the infection is most frequently unilateral, whereas with a generalized infection one would expect a bilateral infection in all cases. The only fact that can be advanced for hæmatogenous origin is the occurrence of rigors and the swinging temperature of the disease, but these only commence with the onset of local kidney signs and symptoms, whereas in a bacillæmia these should be present before the local signs.

The most likely route for the infection is by the lymphatics: either a direct infection from the bowels or from the bladder via the ureteric lymphatics. The last method is not compatible with the clinical picture, as the chief and primary lesion falls upon the pelvis of the kidney, and the bladder is only infected secondarily.

Direct infection from the bowel is simple. On the right side the lymphatics of the cæcum are in direct communication with those of the pelvis of the kidney, while those on the left are more indirectly connected with those of the descending colon. This accounts to a large extent for the greater frequency of right-sided infection.

Pathologically, the whole kidney is involved in the lesion, though clinically we speak of a pyelitis. The main lesion falls on the pelvis of the kidney, the mucosa of which is swollen and covered with mucopus. The kidney substance shows various degrees of invasion; in the mild cases the organ is enlarged, tense and deeply congested, while in the more severe cases multiple small abscesses may be found. The pelvis of the kidney may be dilated and the ureter blocked with inspissated pus and debris, giving rise later to a hydronephrosis. The dilatation of the pelvis, however, is most marked in cases of pyelonephritis of pregnancy, and it is to this condition that I want to draw your especial attention. Dugald Baird, of Glasgow, in the Bradshaw Lecture to the Royal College of Physicians last year, brought the pathology and pathogeny of this subject up to date and added much to our present knowledge of this condition. Since then I have hoped for an opportunity to confirm his findings, as I believed them to be correct. Early in the year I had a case of pyelonephritis of pregnancy, which pregnancy I found necessary to terminate by evacuating the uterus at the sixth month of pregnancy. I evacuated the uterus by hysterotomy, and afterwards made a detailed examination of the urinary tract on both sides and fully confirmed Baird's findings. This case I will describe in more detail later.

It will thus appear that the first factor in the production of acute pyelonephritis is a descending infection via the lymphatics to the kidney from the bowel. In the pregnancy and the enlarging uterus we have the second necessary factor. Greenine and various workers have shown that in pregnancy there is always atony of the upper urinary tract, commencing early in pregnancy, probably due to some endocrine deficiency, and this is thought by Baird to be deficient posterior pituitary secretion. This atony affects both of the ureters and pelves equally; it also causes the ureters to be easily compressible, and the compressing agent is the uterus, and the site of compression the pelvic brim. The greater frequency of right-sided lesion was said to be due to the uterus being more frequently twisted to this side, but observations made at Caesarean section have shown that this is not the case; it may not be twisted at all, and is nearly as often twisted to the left as to the right, and when twisted to the left there has been no corresponding dilatation of the left ureter. Baird has advanced the hypothesis that the greater frequency on the right side is due to the more exposed position of the ureter as it crosses the common iliac artery, while on the left it passes parallel with the vessels and is protected by them, and also passes under the sigmoid, which again protects it. Whatever the cause may be, the right ureter is definitely compressed at the pelvic brim, and this was shown beautifully in my case. Above the brim the ureter was the thickness of a finger all the way up to the pelvis of the kidney, and showed up as a ridge beneath the posterior peritoneum, while below it was only palpable. There was some thickening of the left ureter, but not nearly so marked as the right, and there was no demarcation at the pelvic brim. Baird has described kinks, especially at the junction of the upper and middle thirds; these were not present in my case. Such kinks may represent a more advanced stage of the lesion, which is most probable, as his specimens were obtained at autopsy. By pyelography he has shown dilatation of the pelvis and calyces of the kidney, whilst estimation of kidney function has shown definite impairment. On passing a ureteric catheter in the aforementioned case of mine, at the time, a small amount of urine, pus and debris were evacuated, but later there was no drainage from this kidney at all, and on palpating the kidney at operation it felt firmer and smaller than that on the left side. I wondered if owing to the prolonged back-pressure it had ceased to function, but this is hardly likely, as the pressure of the uterus could never cause complete obstruction of the ureter; but this small fibrotic kidney does correspond to some of the post-mortem kidneys found in the condition. I cannot do better than sum up these pathological conditions in Baird's own words:—

"The usual sequence of events in pregnancy is as follows. At the beginning of pregnancy a primary atony of the ureters occurs. In some this is very slight and may be scarcely appreciable. In others it affects both ureters, equally in primigravidæ, but in multiparæ it may be more pronounced on one side, usually the right, where presumably there has been a previous dilatation of a marked degree. At the third month the primary atony disappears. At the fourth to the fifth month delay in excretion occurs, usually more marked on the right side. At this stage the delay may be due to atony without dilatation; but, more commonly, dilatation of the ureter above the pelvic brim can be demonstrated. The degree of stasis is not in proportion to the amount of dilatation present, as considerable dilatation may be present without any stasis. In some cases spasm of the ureter at the pelvic brim can be demonstrated at this stage, causing severe spasmodic pain over McBurney's point. In such cases there is marked activity of the urinary tract, but delay in excretion occurs due to the spasm, and there is dilatation and kinking of the ureter above the site of the spasm. Where dilatation is accompanied by atony pain is usually absent, but if present is slight and of a dull aching character. The spasm of the ureter is probably the result of the pressure of the uterus, as it can be produced by an ovarian tumour of the same size. The stasis and dilatation become more pronounced until they reach a maximum, usually at the sixth month. From then until term elimination slowly improves. On the left side, where the delay usually has been less marked, this improvement, which may be accompanied by a decrease in the amount of dilatation, is easily seen; but on the right side, when there has been very marked stasis and dilatation, there are technical difficulties in the way of appreciating moderate degrees of improvement. Within a very short time after delivery the ureters again show atony, although the excretion time may be improved."

Clinically the disease may be divided into two types, severe and mild.

In the severe type the onset is sudden, often with an initial rigor, and these may be repeated during the course of the disease. In pregnant cases the onset is usually about the fifth or sixth month of pregnancy. The temperature is high, temperatures of 105° F. are common; on the other hand, the pulse is not unduly rapid and does not correspond to the temperature; later in the disease, however, it may become rapid, and is then a disquieting feature. Pain is referred to one or other loin and the lower abdomen, and there may be some rigidity, and right-sided infection is sometimes mistaken for appendicitis. The pain may be paroxysmal in character, and is then referred to the lower abdomen, but usually the pain is fixed and referred to the loin. There is frequency and pain on micturition, and paroxysmal pain may be associated with this.

On examination there is tenderness over the affected kidney and palpation is resisted, but when the kidney can be felt it is usually enlarged and tender. The urine is highly acid, pale in colour, and a deposit of white muco-pus forms on standing, but this is not always the case, the urine may be quite clear owing to blocking of the affected ureter.

Microscopical examination shows the presence of pus, epithelial and granular casts, a few red blood-cells and B. Coli in profusion.

As the case progresses the temperature gradually becomes lower and the pain decreases in intensity, frequency and pain on micturition lessen, and in five to fourteen days the signs and symptoms disappear; but B. Coli may still be found in the urine, and relapse may occur just as one thinks the patient is cured. This is especially seen in pregnancy cases. On the other hand, the case may drag on with severe constitutional disturbance due to toxæmia, the patient becomes emaciated, the pulse rapid, though the temperature may not be unduly high, and jaundice may appear.

In the mild cases there is a dull, aching pain in the loin made worse by movement. The constitutional symptoms are slight, and these cases may pass unrecognized unless a catheter specimen of urine is examined, when B. Coli, pus-cells and casts will be found. These mild cases occurring during pregnancy often give rise to an erroneous diagnosis of toxic albuminuria, owing to the presence of albumin in the urine, the lumbar pain and general malaise, but the blood-pressure is not raised and the blood urea is within normal limits.

Turning to the treatment of these conditions. In acute pyelonephritis rest in bed is essential, the patient should be kept warm and a blanket bed is preferable. Diet should be "milk" and later "light." Fluids should be given freely, barley water or lemonade. The bowels should be kept open by saline purgatives. Medicinal treatment consists in first rendering the urine alkaline by giving 30 grains each of sodium bicarbonate and potassium citrate every two hours until this is accomplished, and then every four hours for seven days. By this time the temperature is usually normal and the acute symptoms have subsided. Now is the time to turn the reaction of the urine back to acid and give a urinary antiseptic, and for this purpose acid sodium phosphate 30 grains and hexamine 10 grains t.d.s. are given. One practical point arises here: the hexamine and acid sodium phosphate, if given in a mixture, often cause sickness, due to giving off free formalin in the medicine bottle, and therefore it is better to give them separately. My usual practice is to give the hexamine in tablet form, and at the same time give a mixture of—

| | | |
|---|---|---|
| Acid Sodium Phosphate | .. .. | 30 grains. |
| Tincture of Hyoscyamus | .. .. | 10 minims. |
| Infusion of Buchu | .. .. .. | To 1 ounce. |

When giving hexamine the barley water and lemonade should be stopped and plain water substituted.

As a complement to medicinal treatment local treatment is carried out, and this is especially indicated in those cases which fail to clear up under the intensive alkali treatment. The bladder is emptied by catheterisation and 2 oz. of 1 per cent. mercurochrome are instilled, and the patient instructed to hold this as long as possible. If dysuria is still present it is better to commence with ½ per cent. mercurochrome to see how this is tolerated. I believe Mr. Walters originated this form of treatment, and has found it to be nearly as efficacious as instillation of the dye into the pelvis of the kidney. I should be glad to hear his present views on the subject.

I have found it to give good results in non-pregnant cases and in the puerperal form, but not in the pregnancy pyelitis, and this is to be expected, as the pregnancy type is associated with atony and obstruction of the ureters.

Lately I have tried pyridium in these cases, and the results have been encouraging. Two tablets of pyridium are given t.d.s. at first, and later this is reduced to one t.d.s.; while giving this the fluid intake must be reduced to the minimum. I have not found any ill-results, even when given during pregnancy, except the production of nausea and vomiting in a certain number of cases. Reduction of the dosage stops this.

In treating pyelitis of pregnancy exactly the same routine is carried out. While in bed the patient is encouraged to lie on the opposite side to the affected kidney, with the idea that it relieves pressure on the ureter.

If the aforementioned treatment fails to cure, then catheterisation of the affected ureter should be carried out. This drains the obstructed ureter and pelvis, and restores the kidney function to normal. By this means many a pregnancy has been preserved which otherwise would have had to have been terminated. If this fails to cure, then some more serious lesion of the kidney should be suspected, such as a suppurative pyelonephritis or calculus.

If all treatment fail, then evacuation of the uterus, as a preliminary to further treatment, must be carried out. If near term induction of premature labour is indicated, but if in the earlier months, then I think the best procedure is hysterotomy. Pyelitis of pregnancy rarely occurs before the fifth month, and evacuation of the uterus at this period of gestation via the vagina is a tedious process, is liable to be attended with mild infection before the fœtus is discharged, and this, as well as the time element, is important, as by the time evacuation of the uterus is decided upon the condition of the patient is usually grave. The uterus having been evacuated, then routine treatment is carried out, usually with rapid success.

*Chronic Pyelonephritis.*

This is more common than one would expect, and is frequently diagnosed as chronic cystitis, with which it is almost invariably accompanied, the nephritic origin of the bladder inflammation being unrecognized. The primary attack usually occurs at some previous pregnancy, and this attack, though apparently cured as far as clinical signs and symptoms are concerned, leaves the patient with a persistent or intermittent bacilluria or some permanent damage to the pelvis or narrowing of the ureter from stenosis. Dodds, in a recent paper, gave the results of re-examination of 79 of 124 patients who had pyelitis during pregnancy on the puerperium, and of these 79, 49 had either a chronic pyelitis or bacilluria alone, that is, over 50 per cent. of the cases. All these cases cleared up under treatment, usually within six months of delivery.

The patient suffering from chronic pyelonephritis is free from symptoms for long periods, though during this time she may pass B. Coli in the urine, and then suddenly she has a subacute attack with pain in the loin, frequency of micturition and dysuria. The constitutional disturbance is slight, and one frequently sees these cases in the out-patient department. On examination there is pain and tenderness in the costo-vertebral angle of the affected side, the kidney is not palpably enlarged and there is no rigidity. There may be some tenderness over the bladder and some pouting of the urethral mucus membrane at the meatus, and sometimes excoriation of the vulva from the highly acid urine.

A catheter specimen shows high acidity, a small amount of pus, usually only on microscopic examination, which also shows the presence of the organism and some epithelial cells and perhaps casts.

On cystoscopic examination the only particular feature is the pouting of the ureteric orifice on one or both sides with a surrounding zone of inflammation.

Under treatment the symptoms subside, but the bacilluria may persist, and repeated subacute attacks are common.

Here we are faced with a much more difficult condition to cure. The usual routine treatment carried out in acute cases will relieve the subacute exacerbations, but a permanent cure by these means is unusual, hence the multiplicity of advertisements for urinary antiseptics which litter our breakfast tables.

In some cases urinary antiseptics, such as hexamine, pyridium, caprikol and hexyl-resorcinol, to mention a few, may affect a cure, but in the chronic cases local treatment of the pelvis of the kidney or kidneys is indicated. The pelvis of the kidney is drained by ureteric catheterisation and the antiseptic instilled into the pelvis ; for this purpose mercurochrome or some silver preparation may be used.

As an aid to treatment vaccine therapy may be tried. An autogenous vaccine is the best, commencing with half a million organisms and working up to 250 million, given at intervals of four to seven days, depending upon the reaction obtained.

The latest addition to our armamentarium is the ketogenic diet, which was elaborated by Clark at the Mayo Clinic. The growth of the B. Coli has been shown to be inhibited by raising the acidity of the urine to a high degree, and this is accomplished by giving a diet in which fat forms the major portion, with a very small amount of carbohydrates and protein. The actual amounts are : fat 250 grammes, protein 78 grammes, and carbohydrate 25 grammes.

Ammonium nitrate 80 grains daily is given orally to increase the acidity. The result of this is to produce a high acidity and ketonuria, which, according to the writer, clears up the condition in about twenty-one days after the production of the ketosis. During treatment the patient should carry on a moderate amount of work or a prescribed amount of exercise, in order to oxidize the fat.

It is obvious from the nature of the treatment that the person must be physically fit to stand this diet, and be able to metabolize this amount of fat; moreover, it is obviously contra-indicated in pregnancy and the puerperium, and it seems to me that it is more suitable for males than females. Personally, however, I have had no experience of this form of treatment, and I should be grateful for more information from others who may have employed it.

I am afraid that I have added nothing new to this subject, but have tried to put before you the present state of our knowledge of this type of infection in the female, more especially that occurring during pregnancy.

